# Developing an educational resource for people experiencing eating disorders during the menopause transition: A qualitative co-design study

**DOI:** 10.1186/s40337-024-01139-0

**Published:** 2024-11-14

**Authors:** Gemma Sharp, Anne Nileshni Fernando, Susan R. Davis, Alisha Randhawa

**Affiliations:** 1 Department of Neuroscience, Monash University, 99 Commercial Road, Melbourne, VIC 3004 Australia; 2 School of Public Health and Preventive Medicine, Monash University, 553 St Kilda Road, Melbourne, VIC 3004 Australia

**Keywords:** Menopause, Eating disorders, Co-design, Lived experience, Education, Resource

## Abstract

**Background:**

The pronounced changes in reproductive hormones, such as oestradiol and progesterone, that occur during the menopause transition can contribute to increased risk of eating disorder onset or exacerbate a pre-existing eating disorder. Despite this heightened risk, there is a lack of available education and support that takes into consideration the unique challenges of experiencing an eating disorder during the menopause transition. This research aimed to qualitatively explore the perspectives of people with a lived experience of an eating disorder during the menopause transition, and to co-design a support option that addressed their unmet needs.

**Methods:**

A Double Diamond co-design process was followed involving four phases: discover, define, develop, and deliver. Seventeen women in Australia with a lived experience of an eating disorder during the menopause transition participated in online workshops across the four phases to identify their unmet health educational needs in experiencing an eating disorder during this transition, develop potential solutions and ultimately deliver a prototype solution in the final phase. All online workshops were recorded, transcribed verbatim and analysed using qualitative thematic analysis. The findings from the previous phase informed the next leading to the prototype creation.

**Results:**

Qualitative thematic analysis identified six major themes across the four phases; lack of awareness of the intersection of menopause and eating disorders, lack of education, limited and stigmatising services, learning from lived experience, resource impact and resource development.

**Conclusions:**

Findings from this study provided preliminary acceptability of a novel online resource to address the unmet educational needs of people experiencing an eating disorder during the menopause transition. Overall positive feedback on the potential for the resource to improve knowledge and empower treatment-seeking was provided by women with lived experience.

## Abstract (plain language summary)

Menopause is the end of reproductive functioning usually marked by the permanent ending of menstrual periods. In the few years leading up to menopause, known as the menopause transition, there can be an increased risk of eating disorder onset or exacerbation of a pre-existing eating disorder. However, there is a lack of available education and support that takes into consideration the unique challenges of experiencing an eating disorder during the menopause transition. As such, this study designed and developed an online educational resource together with and specifically for people experiencing an eating disorder during the menopause transition through a comprehensive series of online workshops. We found that the women in our study were unaware and confused regarding the intersection of eating disorders and menopause. They provided positive feedback on the online educational resource at the end of the study which they reported improved their knowledge and made them feel more confident to seek further support from health professionals.

## Background

Eating disorder (ED) treatment and support options to date have primarily focused on the needs of adolescents and young adults, with those in midlife receiving less attention [1]. Midlife is generally considered to be between the ages of 40 to 60 years [2]. An estimated 3.5% of women in midlife experience EDs, and the vast majority do not seek treatment [3]. Furthermore, those who seek treatment typically experience poorer treatment and quality-of-life outcomes in comparison with youth and young adults [4]. The lack of treatment-seeking among the midlife population may possibly be explained by the lack of tailored services and support available to this age-group. The presentation of EDs among youth and midlife populations are distinct in several ways including prevalence of different ED diagnoses and ED behaviours [4, 5].

A distinct aspect of midlife that may contribute to the higher prevalence of EDs observed in cisgender women and people with ovaries is menopause [6]. Menopause is the end of ovarian and reproductive functioning, typically evidenced by the permanent cessation of menstrual periods in people menstruating before their menopause. Menopause occurs at an average of 48.8 years globally [7, 8]. The time between pre-menopause and menopause is called the ‘menopause transition’ which can start several years prior to menopause. The menopause transition commences with significant change in cycle length (longer or shorter) and often a change in menstrual flow (can be more or less) due to erratic ovarian hormone production [7, 8]. While people often use the term perimenopause to describe this phase, the perimenopause also includes the first postmenopausal year [9]. These hormonal changes are commonly accompanied by an array of psychological and physical symptoms which may drive changes in the way that women and people with ovaries feel and care for their bodies [6, 10].

Menopause has been likened to puberty, another important life stage characterised by distinct changes in hormones and accompanying symptoms [6, 11]. Due, in part, to marked changes in reproductive hormones, but also other major life and perception changes such as the relationship with self, the body and other people, it is well established that puberty is a time of heightened ED risk [12]. It has subsequently been suggested that people experiencing menopause may also be more susceptible to the exacerbation or development of EDs [11, 13, 14]. In support of this proposal, preliminary findings suggest that changes in the reproductive hormones oestradiol and progesterone are associated with heightened disordered eating [15]. Hormonal and lifestyle changes during midlife can also commonly lead to unwanted bodily changes and subsequent body dissatisfaction, which may further increase the risk of ED onset [16, 17]. Overall, emerging literature has suggested some association between menopause and ED risk. However, further exploration of this relationship and the underlying mechanisms are vital.

We need to not only better understand the level of ED risk during the menopause transition and post-menopause, but also to distinguish between overlapping symptomatology. Similar to the menopause transition, it is common for people living with EDs to experience irregular or absent menstruation; this is called functional hypothalamic amenorrhoea (FHA) [18, 19]. FHA is commonly linked to chronic severe stress [18]. Significant and prolonged energy deficits, malnutrition and excessive exercise, all of which can be seen in individuals with EDs, have been associated with the development of FHA [20]. Binge eating behaviours have also been demonstrated to interfere with the body’s insulin-response which in turn can also lead to missed or irregular menstrual periods [19]. FHA is associated with different hormonal changes from those that occur at menopause. Oestradiol is characteristically low in both circumstances, but follicle stimulating hormone blood levels are characteristically low in FHA and often high in the menopause transition and elevated in post-menopause. While symptoms, such as mood changes, often overlap, vasomotor symptoms (hot flushes and night sweats) are highly suggestive of menopause [18]. However, not all women experience vasomotor symptoms. As women typically understand they have reached menopause when their menstrual period has ceased and they experience a change in symptoms, women with FHA due to an ED may find it challenging to determine if and when they have reached menopause. This may interfere with their ability to seek appropriate healthcare.

Although the current understanding of EDs during the menopause transition may be somewhat limited, preliminary evidence has observed a high prevalence of EDs amongst women in midlife whose needs may not always be met by current approaches. As a result, these women and people with ovaries may not be receiving the care they deserve to experience the best quality-of-life possible in their life circumstances. Thus, is it important to explore the experiences of people living with an ED during the menopause transition to guide future research and treatment development. Adopting a Double Diamond co-design approach [21], the current study aimed to firstly investigate the unique needs of people experiencing an ED and menopause concurrently and subsequently aimed to explore and develop a solution to address those identified needs.

## Methods

### Participants

People with who identified as having a personal lived experience (current or past) of an ED (of any type) during the menopause transition were recruited through an emailed newsletter from an ED support organisation in Australia, Eating Disorders Victoria. Participants were asked to consult with their health professional treating teams prior to expressing interest for the study to confirm this *combined* lived experience, whether it be current or in the past. All recruitment materials included a link to a very brief online expression of interest form including demographic questions (age, gender, ethnicity, relationship status) and health characteristic questions (eating disorder diagnosis, last menstrual period timing, menopause status) to be completed by potential participants. Participants who registered with the online form were then personally contacted by a member of the research team and invited to participate in an online workshop based on availability. Seventeen participants in total were recruited. At the commencement of each workshop, verbal consent from each participant was confirmed (after previous written consent was provided). At the conclusion of each workshop attended, participants were reimbursed with a $30 Australian dollar online gift voucher. This project was approved by Monash University Human Research Ethics Committee (ID: 40040).

The 17 participants ranged in age from 33 to 61 years (*M* = 50.4, *SD* = 8.7) and all identified as cisgender women. The majority of participants (*n* = 15, 88%) identified as White ethnicity, with one participant identifying as Asian (6%) and a further as mixed ethnicity (6%). Over half nominated their relationship status as single (*n* = 9, 53%). The most common ED experienced during the menopause transition according to a diagnosis from a health professional and subsequent participant self-report was anorexia nervosa (*n* = 7, 41%), followed by bulimia nervosa (*n* = 4, 24%), binge eating disorder (*n* = 4, 24%) and other specified feeding or eating disorder (*n* = 3, 18%) noting that some participants nominated more than one diagnosis. All 17 participants reported experiencing an ED prior to the menopause transition (i.e., not new onset of an ED), during the menopause transition (in accordance with the study eligibility criteria) as well as at the time of the study. The majority (*n* = 11, 65%) reported no menstrual periods for the last 12 months or more and stated that they were “postmenopausal” according to their discussions with their health professional teams with the remainder (*n* = 6, 35%) reporting a “perimenopausal” status. A range of menopausal stages were purposely included in the study to ensure broad perspectives on needs during the menopause transition.

### Data collection

**Fig. 1:**
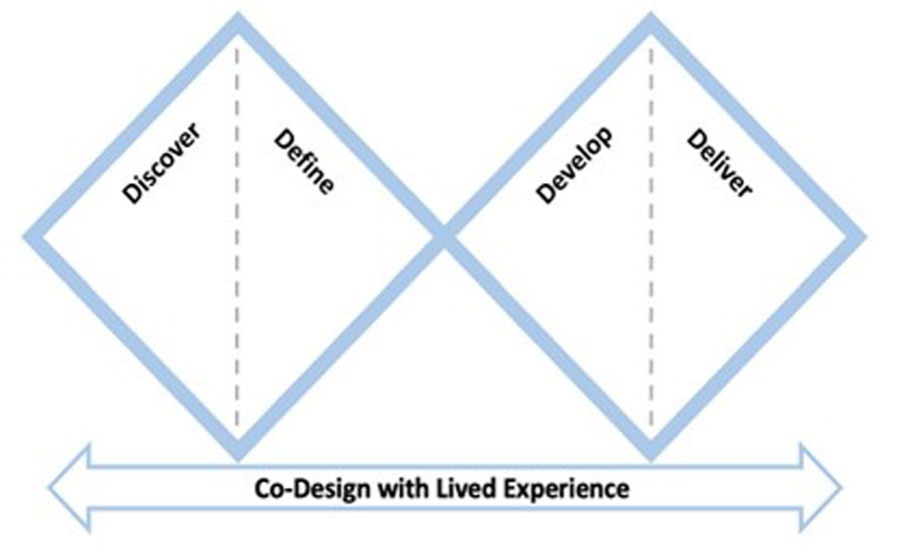
Double diamond strategy adapted from [21]

The Double Diamond approach was implemented to collect co-design data (see Fig. 1). This approach employs four distinct phases that allow for a comprehensive co-design process with several stages of input from people with lived experience [21]. The phases aimed to understand the challenges faced by the affected population (discover phase), characterise innovative ways to address these challenges (define phase), explore how to most effectively implement these solutions (develop phase), and test and refine the proposed solution (deliver phase).

For all phases, data was collected through co-design workshops led by author G.S. and supported by authors A.R. and A.N.F.. The authors developed a semi-structured question guide for each phase which prompted lived experience participants to share their perspectives and feedback on presented ideas. The semi-structured nature of workshops allowed for follow-up questions and discussion, thereby providing more in-depth descriptive data.

Each of the four phases included 9 to 11 participants in total. Two to three workshops were conducted for each phase, with up to five participants in each workshop. New participants were introduced at each of the four co-design phases to ensure a diverse range of perspectives and feedback. All workshops were conducted and recorded through the digital platform Zoom. Phase one workshops (September 2023) lasted from 50 to 63 min (*M* = 57 min), phase two workshops (November 2023) lasted from 61 to 82 min (*M* = 72 min), phase three workshops (January 2024) lasted from 77 to 90 min (*M* = 82 min), and phase four workshops (April 2024) lasted from 64 to 90 min (*M* = 78 min).

### Design process

**Fig. 2:**
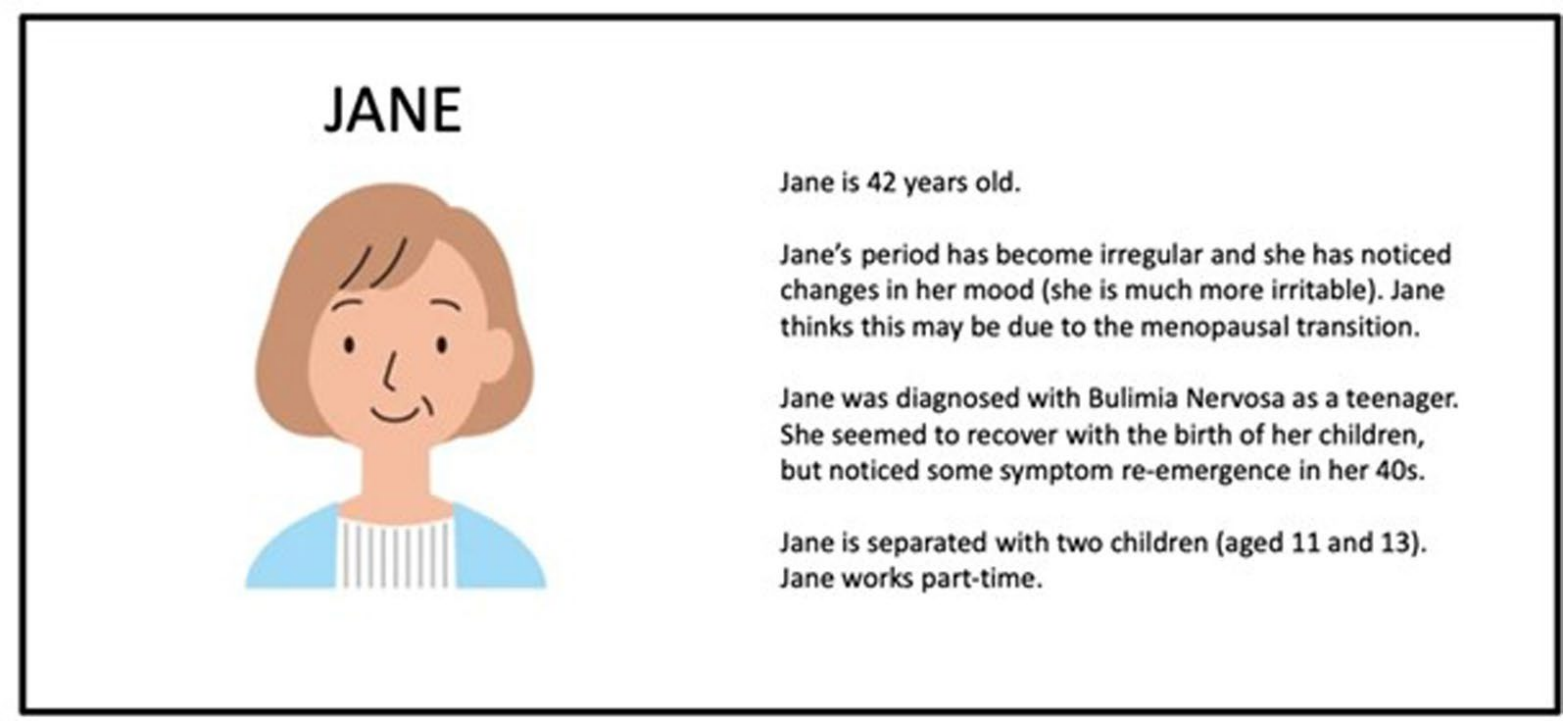
Example persona, “Jane”, used in the develop phase of the co-design process

According to the Double Diamond approach for co-design, the first (discover) phase of workshops aimed to understand the experience of people with an ED during the menopause transition. This included discussion of participant’s individual experiences, factors that were helpful and areas for improvement to better support them during this time. Using key ideas in the first phase, the possibility emerged for an online resource to improve support and address these challenges. The second (define) phase involved brainstorming solutions to address the unmet needs of people living with an ED during menopause, and an online resource was proposed to participants during this discussion. In the third (develop) phase, the feedback from the previous phase was implemented to develop mock-ups of a proposed resource to address the challenges experienced by participants. The mock-ups aimed to provide a model of the proposed resource content and presentation. At the beginning of each workshop in the develop phase, discussion was used to develop a co-designed persona of a typical user of the resource, and feedback throughout the workshop was given through the perspective of the persona (see Fig. 2).

**Fig. 3:**
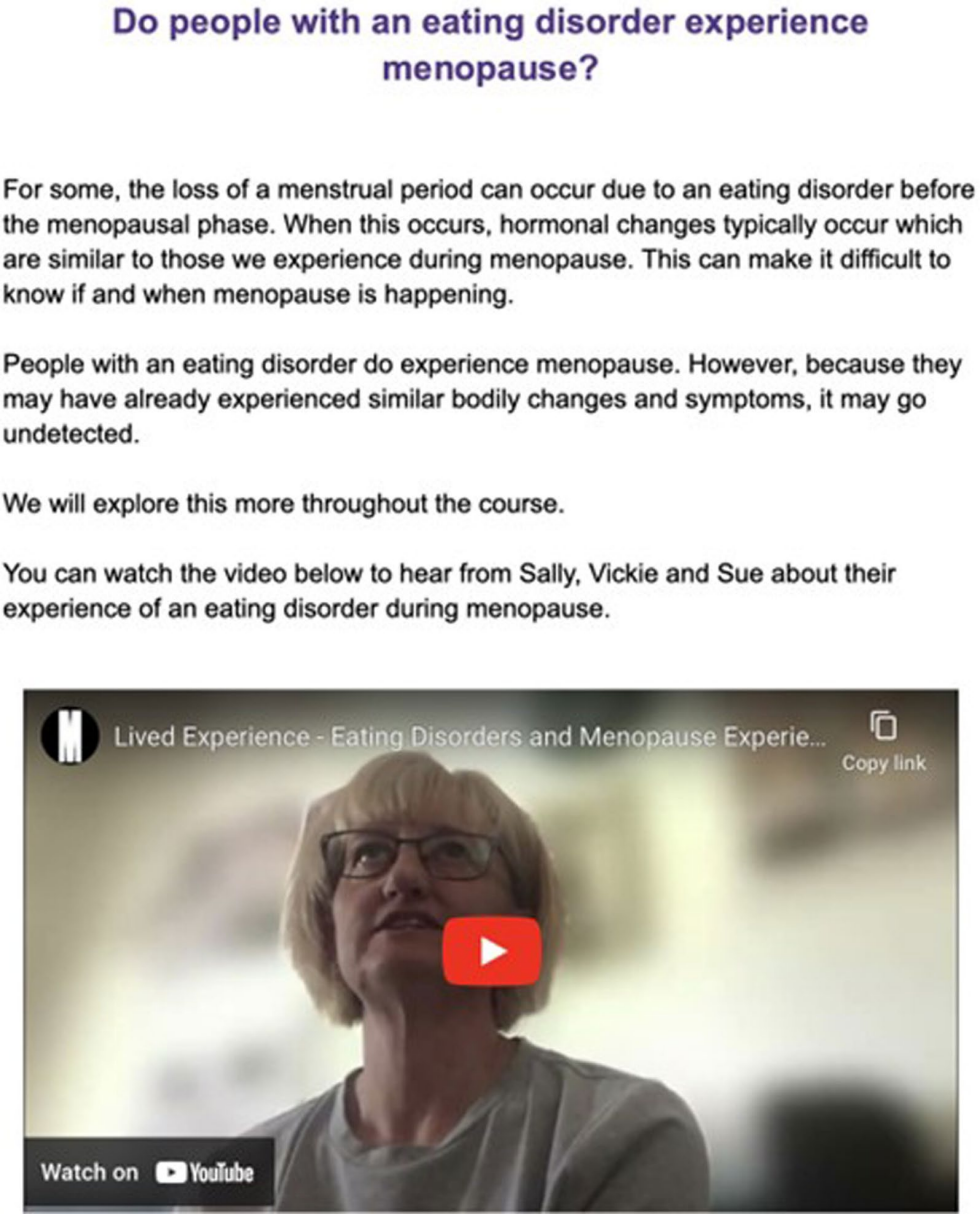
Example of prototype resource content (written and video formats) shown during the deliver phase

The feedback from phase three was implemented before developing a working prototype of the resource. To enhance visibility and user interaction, the resource was developed within the well-established online learning platform of Eating Disorders Victoria, LearnED [22]. The fourth (deliver) phase presented this prototype to participants for final feedback and refinement of both the content and presentation of the resource (see Fig. 3). The final version of the resource can be freely accessed from Eating Disorders Victoria’s e-Learning Portal, LearnED [22] where the following sections are included: (1) Welcome, (2) Eating Disorders, (3) Menopause, (4) Eating Disorders & Menopause, (5) Seeking Support, (6) Helpful Resources, (7) Supporting a Loved One, (8) Information for Health Professionals, (9) About the Team, and (10) Giving Feedback.

### Data analysis

A multi-stage thematic analysis framework [23], led by author A.R. and supported by authors G.S. and A.N.F., was adopted to analyse the data collected from workshops. In stage one, audio recordings for each workshop were transcribed using a confidential automated service, and transcriptions were manually checked for errors and preliminary ideas. In stage two, authors completed another reading of each transcript to manually generate initial codes. Stages three to five of thematic analysis involved collating codes into themes before reviewing and defining these themes. Codes for each individual phase of the co-design were grouped into themes which were used to inform the resource development for the next phase of the study. Once all four phases of the Double Diamond co-design were complete, codes across the entire data set were analysed for overarching themes. The codes and themes were reviewed, refined and named and agreed upon by all authors.

## Results

Data analysis revealed six major themes across almost all phases of the co-design process. These themes included lack of awareness, lack of knowledge, limited and stigmatising services, learning from lived experience, resource impact and resource development; each theme is explored below and illustrative quotes are presented in Table 1.

**Table 1:** Themes and illustrative quotes from all four phases of the co-design process

Theme	Quote number	Illustrative quotes
Theme: Lack of Awareness	1	“God, wow, why aren’t we educated about these things? Because I was very unprepared for perimenopause.” Woman, aged 61, experienced AN and BN, Discover phase
	2	“This is really interesting for me, because it’s never actually been brought up before.” Woman, aged 35, experienced OSFED, Define phase
	3	“We have to empower other women, young people… I talk about this with my daughters because I want them to think about this now, when they’re 26 and they are young, and they are healthy. They need to think about [how] it’s not going to be like this the whole time … be prepared for the change.” Woman, aged 58, experienced BED, Develop phase
	4	“[Eating disorders] are still taboo and even menopause is still taboo. Get this [resource] out to the doctors, to people, to family, to get the conversation started.” Woman, aged 57, experienced AN and BN, Develop phase
Theme: Lack of Knowledge	5	“I feel like it's still not really widely acknowledged…the understanding of women's cycle, their hormone levels, and the impact on their body generally.” Woman, aged 51, experienced OSFED, Discover phase
	6	“It’s incredible how similar the symptoms between eating disorders and menopause can be … is the eating disorder worse because I’ve gone through menopause? Is menopause worse because I’ve got an eating disorder?” Woman, aged 58, experienced AN, Discover phase
	7	“This [resource] is great for us but if the clinicians treating us don’t come on board with the information or awareness, it’s sort of like talking to a brick wall.” Woman, aged 41, experienced AN, Define phase
Theme: Limited and Stigmatising Services	8	“You need more compassion, you need [health professionals] to work with you, to walk with you, and not tell you that you’re an attention seeker.” Woman, aged 53, experienced AN, Discover phase
	9	“It’s interesting to get to this age and still not be taken seriously, or just cast aside … I’ve had doctors say to me that ‘you’re a grown woman and you should know better than this’.” Woman, aged 61, experienced AN and BN, Discover phase
	10	"I think it would be really helpful if there was a broader knowledge amongst staff to be able to implement severe and enduring approaches in inpatient settings like improving quality-of-life. And often they can't, because they're trying to deal with younger patients for whom it isn't appropriate to treat that way … it's too hard for them to deal with the older patients.” Woman, aged 61, experienced AN and BN, Discover phase
	11	“This [resource] is so needed because I feel like you go to the doctor and they’re just focused on the one thing that's wrong with you, and they won't talk about anything else. So, if you're going through menopause and an eating disorder, they might just focus on the eating disorder and throw out the menopause…that's what I found. And then you walk away, having learnt nothing at all.” Woman, aged 54, experienced AN, Define phase
Theme: Learning from Lived Experience		
Subtheme: Diversity	12	"…really making it clear that people's experiences will differ widely… it's not an absolute, there's a lot of unknowns.” Woman, aged 51, experienced OSFED, Discover phase
	13	“I've got a few friends, we're around the same age, doing the same things, and it presents very differently.” Woman, aged 51, experienced OSFED, Develop phase
Subtheme: Lived Experience Voice	14	“[The resource] really makes them walk in your shoes and hopefully empathise a bit with what’s going on. I think there is a big push for health professionals to understand this.” Woman, aged 51, experienced BED, Define phase
	15	“I think it would be really worthwhile to add that … we did draw on people's lived experience in preparing this [resource]. I would feel good if I read it knowing that ‘Ah, okay, so people with lived experience, people like me, have actually helped’.” Woman, aged 58, experienced AN, Develop phase
Theme: Resource Impact		
Subtheme: Education	16	“Me being new to this perimenopause … this [resource] has all the questions that are going through my head.” Woman, aged 35, experienced OSFED, Define phase
	17	“We can understand [the resource] and go ‘Yeah, that's what I'm going through. And that's why I'm going through it’, rather than just going through without any knowledge at all.” Woman, aged 53, experienced OSFED, Define phase
	18	“It's [the resource] great, really comprehensive and just when you're thinking ‘what could that be?’, the definition … [or] ‘click here for more information’.” Woman, aged 58, experienced AN, Deliver phase
Subtheme: Empowerment	19	“If you have this knowledge, when you go to the doctor, you can then put your treatment in the right path, because you're not going in blind with not knowing anything and you can ask for what you want.” Woman, aged 53, experienced OSFED, Define phase
	20	“Women can feel confident in knowing about what's happening to their bodies, so that when they go and see health professionals who don't necessarily understand … they can actually advocate for themselves.” Woman, aged 51, experienced OSFED, Develop phase
	21	“It’s good to know about the Australasian Menopause Society; I didn’t know… being able to find a doctor who would listen would be useful.” Woman, aged 41, experienced BED, Deliver phase
	22	“I feel a leaflet or a handout, something with specific details and instructions for a GP [general practitioner] about testing for hormones properly, so that we can see what's going on, and also working with us to support the fact that we might need something different.” Woman, aged 51, experienced BN and BED, Deliver phase
Theme: Resource Development		
Subtheme: Existing Resources	23	“For me, someone who's had [an eating disorder] for 17 years, I would just get through ‘what is an eating disorder?’ … I'm just sort of sick of reading it.” Woman, aged 33, experienced AN, Discover phase
	24	“Majority of women [are] more familiar with their eating disorders [in that] 40 to 60 year old age group … we want to know more about the menopause, we certainly want to know about the intertwined coining of the two.” Woman, aged 58, experienced AN, Develop phase
	25	“For someone like me who’s gone through a million types of therapies and cognitive behavioural therapy didn’t work, I feel kind of bummed every time I hear stuff about cognitive behavioural therapy.” Woman, aged 33, experienced AN, Deliver phase
	26	“It seems really thorough, and very readable… I haven’t found any gaps, it’s a lot of information which is great.” Woman, aged 58, experienced AN, Deliver phase
Subtheme: Tone	27	“It was an easy read; I could understand it… gets straight to the point and gives you the most important information.” Woman, aged 53, experienced AN, Discover phase
	28	“We don’t want the sugar-coated version.” Woman, aged 40, experienced AN and BN, Develop phase
	29	“That’s reaffirming, that’s nice knowing ‘okay, so I’m not reading this [resource] as a waste of time. They hear me, I’m in the correct place, I’m not being silly.” Woman, aged 58, experienced AN, Develop phase
Subtheme: Design/Presentation	30	“You want information quickly and easily rather than having to read through a lot of text … something that can be represented diagrammatically, or even if you have videos or little audio recordings … it just might make it easier for people to absorb that information.” Woman, aged 51, experienced BED, Define phase
	31	“Have both [text and videos]. Some people will watch one, some people will read it and some people will do both.” Woman, aged 58, experienced AN, Deliver phase
	32	“I love [the videos]. I think they’re very informative.” Woman, aged 51, experienced BN and BED, Develop phase
	33	“That [resource] was actually really easy to take in.” Woman, aged 53, experienced OSFED, Develop phase

*AN* anorexia nervosa, *BN* bulimia nervosa, *BED* binge eating disorder, *OSFED* other specified feeding or eating disorder

### Themes

### Theme: lack of awareness

Most participants reported that they had very little prior awareness of menopause, and its relationship with EDs, across all four phases of the co-design. This perceived lack of preparation for menopause was seemingly driven by menopause being considered a societal taboo topic and a subsequent lack of awareness raising for women at younger ages (Quotes 1–4). This lack of awareness also extended to the greater community, with participants highlighting the importance of improving the understanding of support networks, such as family and friends (Quotes 3 and 4). Participants emphasised the need for a resource to promote the conversation, teaching and normalising of menopause and midlife EDs to improve awareness for all (Quotes 3 and 4). Subsequently, the tone and content of the resource aimed to promote this message, and a section targeting support networks was added to the mock-ups in the develop (third) phase.

### Theme: lack of knowledge

A prominent theme across all phases was confusion regarding the overlap of physical and psychological experiences of the menopause transition and EDs, and difficulties in finding answers to questions on these topics. In the first and second phases (discover and define), participants spoke in depth of their frustration of having no explanation of what was happening in their bodies, why they were experiencing a range of symptoms and how to best address them (Quotes 5 and 6). Adding to their frustration and confusion, participants commonly reported that health professionals were lacking in education and training about menopause transition and concurrent EDs (Quote 7). Participants reported that when seeking answers and support, health professionals were often unable to explain or treat their concerns.

Through the first to third phases (discover, define and develop), participants emphasised that the central focus of the resource must be explaining the overlap of menopause and EDs, including the hormonal changes, symptoms and treatments (Quote 5). It was important to participants that the resource also supported the learning of health professionals (Quote 7). These reports led to the inclusion of two additional sections in the resource during the third and fourth phases (develop and deliver); one that compared the menopause transition and ED experiences, and one that directly addressed health professionals.

### Theme: limiting and stigmatising services

Consistently reported throughout all four phases, participants expressed disappointment and frustration with the lack of services that catered to the needs of people experiencing an ED during midlife and, specifically, the menopause transition. Participants reported that in midlife, they were lacking age-appropriate services that took into consideration their often longstanding EDs (Quotes 9–11). From the first phase (discover), current services were believed to be too heavily focused on alleviating physical manifestations of an ED, such as increasing weight (for underweight participants) and regulating eating behaviours. Participants with longstanding and complex EDs reported minimal interest or hope in trying to alleviate their EDs, and alternatively expressed preference for treatment options to focus more on improving quality-of-life with their ED (Quote 10). Consequently, in the second phase (define), in addition to evidence-based ED and menopause treatment options, exploration of the quality-of-life approach was proposed and content subsequently included in the mock-up (develop) and prototype (deliver) phases.

Across all phases of the co-design process, a recurring theme of feeling invalidated by health professionals and services was evident and that participants should have ‘known better’ by their more advanced age compared to younger people with EDs (Quotes 8–11). Participants consistently reported that their health professionals lacked compassion, and ignored or dismissed their concerns about the menopause transition by attributing all health concerns to their ED diagnosis (Quote 8 and 11). As a result, a section in the resource to directly address health professionals and highlight these concerns was included from the third phase (develop).

### Theme: learning from lived experience

#### Subtheme: diversity

The extensive variety in people’s experiences of EDs, menopause and their overlap were emphasised throughout all phases. From the first phase (discover), participants highlighted how their experiences, including symptoms and feasible support options, differed greatly (Quotes 12 and 13). Thus, in all subsequent phases (define, develop and deliver), it was consistently reported that the resource must showcase this diversity in experiences and offer information that catered to all as much as possible (e.g., information for people experiencing “early menopause” around the age of 40 [8]). Consequently, frequent messages of diversity were included in the final resource and stereotypical experiences were deemphasised.

#### Subtheme: lived experience voice

In fostering this message of diverse experiences, the value in hearing and learning from people who had experienced an ED during the menopause transition was frequently discussed throughout all phases of the co-design (Quotes 14 and 15). In the first phase (discover), when discussing what was helpful in participants’ experiences of menopause and EDs, connecting and learning from peers was consistently reported. Furthermore, participants emphasised how the value of learning from diverse lived experience should also be applied to health professionals (Quote 14). Consequently, across the second to fourth phases (define, develop and deliver), a number of additions were proposed and implemented to the resource to foster the voice of lived experience. These additions included explicitly stating the contribution of participants in the development of the resource, interspersing video and written lived experience stories throughout the resource, and catering the resource to health professionals in addition to people experiencing an ED during menopause to ensure that all stakeholders were learning from lived experience.

### Theme: resource impact

#### Subtheme: education

In response to the lack of knowledge reported by all participants, it was highlighted in the first and second phases (discover and define) that the resource must provide answers and address confusion about the overlapping experience of EDs and menopause transition (Quotes 16 and 17). In the third and fourth phases (develop and deliver), participants expressed positive feedback that the information (text, diagrams, videos) included in mock-up and prototype versions of the resource were comprehensive and answered their questions about what happens in the body during EDs and menopause and how these can be treated (Quote 18).

#### Subtheme: empowerment

In the second phase (define), when aiming to conceptualise if and how an online resource could address participant’s previously reported concerns from phase one (discover), it was reported that a resource could equip people experiencing an ED during the menopause transition with the knowledge and pathways to seek treatment and support further learning. Participants reported feelings of excitement and motivation to learn more, and that they could use the information they learned from the resource to advocate for their treatment with their health professional teams (Quote 19). Fostering this goal of empowerment, feedback from the third phase (develop) included that the resource encouraged further reading and learning, provoked action and would help with self-advocacy during treatment seeking (Quote 20). The resource was further reported (deliver phase) to connect participants with treatment and support pathways of which they were not previously aware (Quote 21). To consolidate and optimise this sense of empowerment in the fourth phase (deliver), participants requested a help-seeking guide/tips to follow when treatment seeking (Quote 22). Participants wanted further support on the information they should present to their health professionals (particularly in primary care settings) and the medical tests/support options to be explored. This guide was developed and included in the final resource.

### Theme: resource development

#### Subtheme: existing resources

In the first phase (discover), participants expressed the need for information and support that was tailored to the overlap and combined impact of EDs and menopause. When discussing the potential feasibility of an online resource, it was clearly emphasised that there was an abundance of existing ED focused resources, and another would be unnecessary and unhelpful for their particular age group and life stage (Quote 23). This extended to the inclusion of information about psychological therapies, particularly enhanced cognitive behaviour therapy (CBT-E), which all participants stated had been an ineffective form of treatment for them despite being the first line treatment for adults with EDs (Quote 25). Across the second and third phases (define and develop), participants continued to emphasise that the resource should target the association between EDs and the menopause transition (Quote 24), rather than exploring them separately. This feedback resulted in the creation of an additional resource section that addressed the overlap of EDs and menopause during the third phase (develop). In the fourth phase (deliver), participants did not continue to emphasise the need for information on the overlap, rather reporting that the resource was comprehensive and informative (Quote 26). It must be noted that while participants tended to express that they were not interested in the introductory sections of the resource that defined EDs and menopause separately, a shortened version of this information remained in the resource to ensure that all people accessing the resource, who may not have prior knowledge, had access to all the relevant information.

#### Subtheme: tone

From the earliest phases of the co-design process, participants reported that it was important for the resource to feel warm and inclusive, hopeful, authentic and straightforward (Quotes 27 and 28). In the later phases, participants reported that they felt the resource was sympathetic and friendly, gave them hope and encouragement, was honest and truthful about the experience of EDs during the menopause transition, and was easy to understand (Quote 29).

#### Subtheme: design/presentation

Across all four phases of the co-design, participants demonstrated differing opinions on how information should be presented to cater for different learning styles. To account for this diversity, information was presented in the resource using a combination of written, image, video and audio formats (Quotes 30 and 31). In the final phase (deliver), participants reported that the resource did not include too much text, and the image, video and audio content was engaging, helpful and easy to understand (Quote 32 and 33).

## Discussion

The current study aimed to investigate the unique needs of people living with an ED during the menopause transition, and co-design a strategy to address these potentially unmet needs. Through exploration of lived experience reports, a lack of available information, awareness, and accessible support options was evident. Consequently, the value of an online, informative resource was considered, discussed and co-developed. To the best of our knowledge, this is a highly novel resource that comprehensively explores the intersection of EDs and menopause. Analysis of the qualitative data identified six major themes across the four phases of this study; lack of awareness, lack of knowledge, limited and stigmatising services, learning from lived experience, resource impact and resource development. Based on the overall positive feedback provided from participants with lived experience, the resource was deemed preliminarily acceptable.

The topics of menopause and EDs have received relatively less attention compared to other physical and mental health conditions and this may help to explain the seeming lack of available information. The women in our study agreed that people experiencing the menopause transition and an ED concurrently first need the available tools to educate themselves, in order to feel confident in advocating for their own treatment. Most women in our study had commonly experienced irregular or completely absent menstrual periods in their lives, potentially due to FHA [18, 19], and so participation in our study was the first or one of the first opportunities for them to understand the biological mechanisms underpinning their menopause experiences. This notion aligns with previous research that has demonstrated the importance of psychoeducation in the treatment of midlife EDs [1]. Education can not only help patients understand what is happening in their bodies, but also provide them with direction for recovery, and foster attitudes of hope and perseverance [1]. Overall, an informative resource was first encouraged, then well-received by the women in our study who reported that having access to this information could both educate and empower them.

While the resource may support women’s education and motivation, another key concern identified was stigma. The women reported being “dismissed” by health professionals when seeking treatment for an ED during midlife. They added that some health professionals considered EDs to be an issue only impacting younger people and that people in midlife and older should have already recovered. Such reports are consistent with previous research, where people in midlife with EDs have reportedly been met with a lack of compassion and disapproval from clinicians [1, 24]. There have been several calls for health professionals to adopt a sympathetic and gentle approach to ED treatment (e.g., [25, 26]), however our results suggest that there is still some progress to be made in supporting people in midlife with EDs, particularly those in the menopause transition.

It is possible that this “dismissal” of patients by some health professionals may be motivated by a lack of specific training to treat patients in midlife. The consistent reports that health professionals do not have extensive knowledge about menopause and menopause management aligns with previous research [27]. Hence, it is not surprising that the women in our study reported uncertainty by health professionals regarding how to treat EDs and menopause symptoms concurrently. Previous research has found that stigma and lack of compassion from health professionals was exacerbated by clinicians feeling ill-equipped to treat ED patients and being disheartened by patients seemingly not improving [28]. Thus, a lack of hope and uncertainty may be heightened when treating midlife patients who may have a longstanding history with EDs, and for whom there are potentially less treatment options available. Despite the previously identified need for midlife-specific ED services [1, 5], the limited training for health professionals as well as age-specific services are seemingly still felt by some people experiencing an ED during menopause. The resource developed in this study included brief information addressed to health professionals specifically, in an effort to bring awareness to this issue and offer ways to improve the patient experience during service provision.

While current approaches to midlife EDs are not necessarily age-specific, severe and enduring or longstanding ED treatment options can be applied. Psychological therapies such as CBT-E and Interpersonal Psychotherapy are often endorsed in both literature and practice [1, 29]. Interestingly, the women in our study emphasised that these treatment options were usually ineffective in their experiences, and they cautioned about including them in the resource. However, these options were still included in the resource and their research evidence base described to ensure that this information was available to all resource users. Alternatively, the women in our study endorsed a quality-of-life approach that focused on life enjoyment and fulfilment as opposed to symptom recovery. This approach has been found to be preferred by some patients [26] as well as some suggestion of efficacy [30], however more research is needed. Owing to our participant reported preference, in addition to mention of traditional psychological therapies that take precedence in literature, the quality-of-life approach was also described in the developed resource. These findings further highlight the need for the training of health professionals to ensure that they adequately cater for the midlife population and the need for further research investigating a quality-of-life approach.

Additional aspects of delivering an effective resource were also considered in the current study. The women reported the need for an easily accessible, understandable and engaging resource that fostered hope, understanding, inclusion and honesty. A hopeful and empathetic tone has similarly been reported in the co-design of other websites targeting women’s health issues (e.g., [31]). Furthermore, the co-design of a mental health-based website targeted towards Australian adults also found that straightforward, empowering and honest language was preferred, and the use of both visual and written content was important to optimise engagement and comprehension [32]. Therefore, the tone and design aspects adopted in our resource aligned with website development on other sensitive health topics.

## Future directions

The co-design process of this study identified several areas for future research. Firstly, although the developed resource demonstrated preliminary acceptability, the need for further evaluation is necessary. An evaluation survey has been included in the final online resource [22] to ensure real-world users can provide feedback and allow for this further evaluation and refinement of the resource. Secondly, it is clear that specific training for health professionals on the co-management of EDs and menopause is vital. This may address both the stigma of midlife EDs and the efficacy of current support options.

## Limitations

There were several limitations in the present study that should be noted. Firstly, participants were recruited through ED, as opposed to menopause specific or other types of channels. Consequently, participants had high pre-existing levels of ED knowledge which may limit their representation of the wider population. Other people experiencing an ED during the menopause transition may not have such extensive prior knowledge and experiences of EDs and may have different needs from those identified in this study. Nevertheless, we ensured that fundamental information about both EDs and menopause were included in the resource to promote broad understanding. A further participant-related limitation was that all participants had experienced an ED prior to the menopause transition rather than new onset and an individual with new onset may have rather different experiences. Future research should aim to specifically include these perspectives. Another limitation was that some of the demographic characteristics of the sample lacked diversity; participants were all cisgender women of predominantly White ethnicity. This further limits the representative nature of the current sample. Moreover, the demographic and health characteristic information collected from participants was very brief and we relied upon participants discussing their study eligibility (i.e., a current or past experience of an ED during the menopause transition) with their health professional teams rather than asking for additional written information or hormonal testing. We employed such a strategy to keep participant burden of involvement in the study to a minimum and predicted that conversations with health professional teams may potentially be more enlightening for participants given that irregular or absent periods can be common in people experiencing EDs and hormonal testing may not always provide insights into menopause stage [8]. Lastly, whilst the participants reported that the developed resource was informative and promoted treatment-seeking, this is yet to be formally researched. Further investigation is needed to measure the extent to which the resource improves knowledge and empowerment of women experiencing an ED during the menopause transition.

## Conclusions

The current study explored the co-design and preliminary acceptability of an online resource to address the identified unmet health educational needs of people experiencing an ED during the menopause transition. To the best of our knowledge, this is the world’s first comprehensive resource to explore the intersection of EDs and menopause. Women with lived experience provided overall positive feedback on the potential for the resource to improve their knowledge and empower them to seek treatment. If efficacy is demonstrated, this resource may improve awareness and understanding of the ED risk during the menopause transition, and allow people to more confidently seek support.

## Data Availability

The datasets generated and analysed during the current study are not publicly available under the participant confidentiality conditions of ethics approval from the Monash University Human Research Ethics Committee.

## Abbreviations

AN: Anorexia nervosa
BED: Binge eating disorder
BN: Bulimia nervosa
CBT-E: Enhanced cognitive behaviour therapy
ED: Eating disorder
FHA: Functional hypothalamic amenorrhoea
OSFED: Other specified feeding or eating disorder
